# Occurrence of the Toxin-Producing *Aspergillus versicolor* Tiraboschi in Residential Buildings

**DOI:** 10.3390/ijerph13090862

**Published:** 2016-08-31

**Authors:** Marlena Piontek, Katarzyna Łuszczyńska, Hanna Lechów

**Affiliations:** Department of Applied Ecology, Faculty of Civil Engineering, Architecture and Environmental Engineering, Institute of Environmental Engineering, University of Zielona Góra, ul. Prof. Z. Szafrana 15, 65-516 Zielona Góra, Poland; M.Piontek@iis.uz.zgora.pl (M.P.); H.Lechow@iis.uz.zgora.pl (H.L.)

**Keywords:** *Aspergillus versicolor*, sterigmatocystin (ST), moulds, residential buildings, biotests

## Abstract

In an area representative of a moderate climate zone (Lubuskie Province in Poland), mycological tests in over 270 flats demonstrated the occurrence of 82 species of moulds. *Aspergillus versicolor* Tiraboschi was often encountered on building partitions (frequency 4: frequently). The ability to synthesize the carcinogenic sterigmatocystin (ST) means that it poses a risk to humans and animals. Biotoxicological tests of biomasses of *A. versicolor* were conducted in the Microbiological and Toxicological Laboratory, using the planarians *Dugesia tigrina* (Girard). The obtained results of the tests covered a broad range of toxicity levels of isolated strains: from weakly toxic (100–1000 mg·L^−3^) to potently toxic (1–10 mg·L^−3^). The high-performance liquid chromatography (HPLC) physicochemical method confirmed the ability of *A. versicolor* strains to synthesize sterigmatocystin. All of the samples of the air-dry biomasses of the fungi contained ST in the range between 0.03 and 534.38 mg·kg^−1^. In the bio-safety level (BSL) classification *A. versicolor* belongs to category 1. Additionally, *A. versicolor* is an allergenic mould.

## 1. Introduction 

The problem with the occurrence of moulds in buildings is not only the biodeterioration of technical materials, but also the deterioration of the sanitary condition of the indoor air [1,2]. This real risk to the health of residents of infected rooms occurs due to the ability of the majority of species of moulds to synthesize secondary metabolites—mycotoxins. The problem is known and has become more and more serious in recent years, not only in Poland [3,4,5,6]. Based on tests conducted in a residential environment, greatest attention is paid to the presence, development, and forecasting of the development of xero- and hydrophilic moulds, including mycotoxigenic ones, with such dangerous species as *Aspergillus versicolor* and *Stachybotrys chartarum* [7,8,9,10]. Bloom et al. [11] showed that several mycotoxins synthesized by *S. chartarum* (macrocyclic trichothecenes, trichodermin, and satratoxin G) and *A. versicolor* (sterigmatocystin) may be present in the majority of samples coming from the building materials of damp flats or from samples of settled dust. 

*Aspergillus versicolor* is a saprotrophic fungus, which is widespread in nature. It is often present in soil (rotting plants, litter, straw, and the rhizosphere of many plants), on many rotting food products (cheese, peanuts, dry meat products, flour), and on cereals. It produces proteolytic enzymes and often appears on fats and their derivatives, as well as on bread. It also tolerates a very broad pH range, including an alkaline environment. The optimum temperature for its growth amounts to 20 °C–30 °C (min. 4 °C, max. 40 °C). Conidia germinate at temperatures between 12 °C–37 °C [12,13,14]. The fungus is pathogenic (BSL 1) and causes aspergillosis in pet animals [15]. Spores of *A. versicolor* are easily inhalable [16,17]. Their diameters range between 2 and 3.5 µm [14]. They are often detected in lung tissues during an autopsy [17].

*Aspergillus versicolor* is a primary coloniser capable of growing at a_w_ > 0.65–0.70 [14,18,19]. This causes this species to be one of the moulds which is most frequently found on building materials inside premises [20,21,22,23,24,25]. Additionally, it is frequently present in the air in substantial quantities. It colonises air-conditioning equipment and is present in the carpet dust of damp, confined premises. The mould often occurs (frequency 4) in buildings in the Lubuskie province [22], and its proven ability to synthesize sterigmatocystin on building materials was the reason for the initiation of the toxicological tests [10,26].

*Aspergillus versicolor* synthesises important toxic metabolites: 5–methoxysterigmatocystin and dihydroxy–sterigmatocystin, 5,6-dimethoxysterigmatocystin (with cytotoxic properties), averufin, deoxyaverufin, cyclopiazonic acid, nidulotoxin, versicolorin A, B, and C (cytotoxic, mutagenic, and teratogenic), versiconol (with low toxicity), and sterigmatocystin (ST) (ST is the precursor of AFB_1_ and, just like aflatoxin, it is characterised by toxicity and carcinogenicity) [13,17,27,28,29,30]. ST in test animals causes liver necrosis at doses ranging between 18 and 100 mg·kg^−1^ of body weight, and kidney damage at higher doses. Although sterigmatocystin may initiate liver cancer in rats and cause the development of tumours at the site of its administration, its carcinogenic effect is ten times smaller than that of AFB_1_. The toxic effect is also smaller [17]. *A. versicolor* consistently produces carcinogenic sterigmatocystin on the majority of laboratory media. It synthesises substantial quantities of ST and 5 methoxy-sterigmatocystin on building materials [31]. The tests conducted on isolates of *A. versicolor* from the air inside the rooms proved that these strains produced ST in laboratory cultures, and the extracts of the cultures demonstrated cytotoxicity. It was determined that conidia of two strains of *A. versicolor* comprised 0.375 and 1.060 pg (10^−12^ g) of ST in the spore [17]. In laboratory conditions, the strains isolated from the building materials and surface finishes from damp buildings produced ST on gypsum boards, chipboards, and especially large quantities on wall papers [32]. Other strains produced 5 methoxy-sterigmatocystin. The lethal concentrations (LC 50) of ST for shrimp larvae (*Artemia salina*) are 5.95 mg·L^−3^ and 0.54 mg·L^−3^ [33], respectively. The toxicity of ST for the test animals expressed in lethal doses (LD 50 in mg·kg^−1^) amounts to: ducklings, 1.0; albino rats, 166 (males, *p.o*.); albino rats, 60 (males, *i.p*.); albino rats, 120 (females, *p.o*.); albino rats, 65 (females *i.p.*); male monkeys, 32 (*i.p.*) [34].

Volatile metabolites (MVOCs) produced by *A. versicolor* include 2-ethyl-1-hexanol, 1-octen-3-ol, 3-octanone, 2-heptanone, 2-pentanone, 2-hexanone, and 2-methylfuran. Isolates produce pronounced earthy volatiles [35]. 

## 2. Materials and Methods

The paper presents the results of research conducted for many years (more than 20 years), with regards to the occurrence of moulds in residential housing, as well as the assessment of potential risks (allergies, mycoses, mycotoxicoses) and real risks (synthesis of sterigmatocystin, *Aspergillus versicolor*, and also the results of biotests from extracts of *A. versicolor* biomasses), which indirectly confirm the health risk for people living in mouldy premises. The research was conducted in the area of the Lubuskie province in Poland. The area is representative of many buildings erected within the moderate climate zone. Collection of the big number of results (82 mould species, over 270 buildings) allowed the establishment of the frequency of occurrence of the respective taxons on building partitions, as well as the indication of 10 species which colonise walls most frequently. The results, after taking into consideration the potential risks gathered on the basis of many different published references, constituted the grounds for the selection of the species (out of 10 species on the list) for mycotoxicological tests (high-performance liquid chromatography (HPLC) and biotests). *Aspergillus versicolor* became our choice as it is often present in residential housing and consequently synthesises sterigmatocystin, which is a carcinogenic substance from category 2B (according to International Agency for Research of Cancer (IARC) [36]), on the walls. 

Forty-five air-dry biomasses of *A. versicolor* obtained in laboratory cultures and coming from buildings were subjected to toxicity tests. The biotoxicological testing of methanol extracts from mould biomasses was carried out using *Dugesia tigrina* (Girard)*.* Forty-five mould samples were analysed for the presence of sterigmatocystin (ST) in the Department of Pharmacology and Toxicology of the National Veterinary Research Institute in Puławy, using HPLC. Mycological and mycotoxicological tests have been carried out in the Microbiological and Toxicological Laboratory at the Department of Applied Ecology, Institute of Environmental Engineering, University of Zielona Góra (Poland). 

### 2.1. Mycological Tests—Collection of Samples from Buildings, Method of Culturing Moulds

Moulds were collected in over 270 buildings from the inner surfaces of building partitions with visible mould. The partitions were made of plasterboard, bricks, and breeze blocks, and the finishing materials included plaster, acrylic paint, wallpaper, and others. The samples were collected directly at the site of occurrence and transferred onto Petri dishes, which contained such media as malt extract agar (MEA), Merck and Czapek–Dox (Cz), and Merck. Mycological analysis was performed in accordance with the methodology established by CBS (Centraalbureau voor Schimmelcultures) [14]. The direct plating method was used. According to this method, small fragments of the material infected with moulds are transferred or spread onto the Petri dishes containing the culture media [37]. The samples were incubated in a cultivation room, covered with white linen, at room temperature, between 18 °C and 22 °C, maintaining the circadian rhythm of day and night. Pure (axenic) cultures were isolated from mixed starter cultures by their passaging on the media: Cz and MEA. The time of plating, cultivation and observation for an isolated single species was 21 days [38,39]. The isolated strains were subjected to identification tests using keys for taxonomic identifiers: [12,14,15,19,26,40,41,42,43,44,45,46]. Bacteria grew out in the colonies and were not the subject of research.

### 2.2. Acarological Analysis—Collection of Samples from Buildings, Method of Culturing Mites

Samples of mites (*Acari*) were taken directly from the place where moulds were present onto surfaces with two media (Cz and MEA), using Petri dishes. Mites were mounted in a Hoyer medium on slides, and the species and life stages were determined with the aid of an Axioscope 2 Zeiss compound microscope (Carl Zeiss Inc., Oberkochen, Germany). Solarz [47] conducted acarological analysis in Department of Parasitology, Medical University of Silesia in Katowice, Sosnowiec, Poland. 

### 2.3. Cultures of Aspergillus versicolor 

Mass cultures of *A. versicolor* were established for biotoxicological and physicochemical testing of isolated strains. Five millilitres of MEA was poured out on ø 9 cm Petri dishes. The samples were incubated at temperature, between 18 °C and 22 °C, maintaining the circadian rhythm of day and night. The cultivation time for an isolated culture was three months. This is the time required for growth, sporulation, and regrowth until a large area of the Petri dish is covered, and also strain aging and drying of the mycelium with the medium to an air-dry state. According to Piontek [10], the method of the cultivation of the moulds constitutes the simulated environmental conditions prevailing in the various seasons of the year on building partitions in residential housing. The air-dry moulds with the media were collected from the dishes by means of a scalpel, then they were weighed and placed in glass jars closed with a ground stopper. The material prepared in this way served the purpose of the preparation of methanol extracts for biotoxicological tests, as well as test portions for chromatographic analyses with reference to the presence of sterigmatocystin. 

### 2.4. Methanol Extracts for Biotoxicological Tests 

In order to carry out 45 toxicity analyses of *A. versicolor* using *D. tigrina* as the test organism, methanol extracts from the culture of strains isolated in the residential housing were prepared. Mycotoxins were extracted from the mould biomass using the method developed by Piontek [10,38,39]. One-gram air-dry samples of mould biomass were extracted over 96 h in 100 cm^3^ of 80% methanol (analytical grade) before their filtration through Whatman 4 blotting paper placed on a filter flask. In the applied procedure, 1 mL of extract is obtained from 10.0 mg of sample (mould plus medium). 

### 2.5. Biotoxicological Tests Using Dugesia Tigrina 

The toxicity of 45 strains of *A. versicolor* isolated from flats was investigated. For all of them, toxicological tests using *Dugesia tigrina* were carried out. The obtained results were the basis for the calculations of a lethal concentration LC 50, (mg·L^−3^) for test organisms. The method of the cultivation of the planarians for toxicological tests was developed by Piontek [48]. The toxicological tests were performed in a laboratory at temperatures ranging between 18 °C and 22 °C. A series of concentrations was prepared. Test solutions with volumes of 40 cm^3^ were poured into beakers with capacities of 50 cm^3^. 10 cut specimens were introduced into each beaker [10,49,50,51,52]. The determinations were carried out in three repetitions including control tests. Thirty planarians were kept in each concentration of the toxicant. After 240 h, the mortality of the planarians was checked. The obtained results constituted the basis for the calculation of a lethal concentration (240 h LC 50). The graphic interpretation method (probit analysis) was used to calculate the value of the concentrations—LC 50. The obtained results were subjected to the test of compliance of experimental distribution with a normal distribution. The χ^2^ test was used in the calculations. The tested distributions were considered sufficiently convergent with the normal distribution, if the likelihood in the χ^2^ test was higher than 0.7 [53]. The obtained results of the biotoxicological tests of substances for the planarians were evaluated in terms of the degree of their toxicity based on the Liebmann classification [54] (see Table 1). 

### 2.6. The Determination of Sterigmatocistin Concentration by HPLC 

Forty-five air-dry biomasses of *A. versicolor* were tested in the Department of Pharmacology and Toxicology of the National Veterinary Research Institute in Puławy, using HPLC for the presence of sterigmatocystin (ST). The determination of the content of sterigmatocystin in fodder and mycelium was conducted by means of high-performance liquid chromatography (ZFT/PB/06-12/edition 1, date of issue 2011.04.28) [55]. A detailed description of the HPLC method is presented in the Piontek’s paper [38,39].

The toxicity of ST was evaluated based on the classification established by Chełkowski [13,33], (Table 2). 

## 3. Results 

### 3.1. Results of Mycological Tests 

In the mycological tests conducted in over 270 flats, 82 species of moulds (Table 3) were isolated from walls with visible mould.

The presence of two or more species of moulds in one sample was confirmed in reference to all of the tested samples. As well as the moulds, the presence of numerous bacteria and allergenic mites (*Acari*), belonging to *Tyrophagus putrescentiae* was found [10,47,56]. For isolated species of moulds the frequency of their occurrence on the building partitions was determined. Scale 1–5: >0%–5% (1, sporadically), >5%–10% (2, individually), >10%–15% (3, fairly frequently), >15%–20% (4, frequently), >20% (5, very frequently) according to Piontek [22]. 

The following species of moulds were present with the highest frequency: *Cladosporium herbarum, Mucor racemosus*, and *Penicillium chrysogenum* (frequency 5, very frequently). Two species were present frequently: *Ulocladium chartarum* and *Aspergillus versicolor* (frequency 4, frequently). 

### 3.2. Toxicological (Biotoxicological and Physicochemical) Tests

The specification of the obtained results of physicochemical and biotoxicological tests is presented in Table 4. 

As the obtained results of the biotoxicological tests indicate, 30% of the strains of *A. versicolor* were weakly toxic (Class IV, according to Liebmann’s classification), 26 strains (58%) belonged to Class III (medium toxic), for which the LC 50 ranged between 10 and 100 mg·L^−3^, and six strains (13%) were potently toxic (Class II). At the National Veterinary Research Institute in Puławy, the analysis was performed based on 45 biomasses of *A. versicolor* (Table 4). The quantity of sterigmatocystin contained in the tested biomasses ranged between 0.03 and 534 mg·kg^−1^ (Table 4). *D. tigrina* turned out to be a sensitive bio-indicator for the presence of ST, which is confirmed by the quantity tests performed using the HPLC method. Nine tested biomasses demonstrated the presence of ST above 100 mg·kg^−1^ (24%), which classifies these strains as very potently toxic, 51% of biomasses were potently toxic (the quantity of ST ranged between 13.6 and 99.7 mg·kg^−1^). 20% of biomasses were weakly toxic, whereas four biomasses of *A. versicolor* with the quantity of sterigmatocystin below 1 mg·kg^−1^ were non-toxic (n.t.).

## 4. Discussion

In the presented test results, as well as results presented by other authors in papers concerning this subject, it has been proved that the moulds synthesise mycotoxins (secondary metabolites) on building materials [21,27,31,32]. The more relevant ones for the selected species of moulds occurring with high frequency in the residential housing in the area of the Lubuskie province are listed in Table 5. 

The risk of the release of mycotoxins may occur when spores and fragments of moulds, as well as particles containing mycotoxins such as dust or mouldy substances, are inhaled and swallowed. Therefore, the most important issue is to determine which, as well as how often and how many, mycotoxins are present naturally in mouldy building partitions in residential housing [10]. The toxigenicity trait of moulds is not a stable phenomenon [57]. It is a fact that certain species have mycotoxin-positive strains, and others do not have the ability to synthesise mycotoxins. Therefore, after the isolation of moulds from the building materials, they must be tested in terms of their ability to synthesise mycotoxins by performing an analysis of the biomasses of isolates obtained from laboratory culture on microbiological media [10,38,39].

*A. versicolor* is the main producer of carcinogenic sterigmatocystin with nephrotoxic, hepatocarcinogenic and immune-suppressive properties. The tests performed in the Microbiological and Toxicological Laboratory confirm that *A. versicolor* is a species of mould which may constitute a risk to residents of infected rooms. It is often present on the building partitions of buildings located in the areas representative of a moderate climate (Table 3). The toxicological tests for *D. tigrina* demonstrated the occurrence of *potently toxic* strains (13%), which means that the biological methods are applicable for the evaluation of the mycotoxic risk in buildings (Table 4). The various quantities of ST, including nine strains which produced more than 100 mg·kg^−1^ of it (*highly toxic* strains according to Chełkowski) justifies the need to conduct research on this species. Chemical analysis unfriendly to the environment could be replaced by biological one. Physicochemical analysis (HPLC) should be conducted in the case of a high toxicity level of the sample. 

Tests of dust from damp residential premises conducted in Germany demonstrated the occurrence of the species of mould called *A. versicolor*. The majority of the strains (98%) of *A. versicolor* isolated from the dust was able to synthesise ST in vitro [23]. Mycological tests conducted in Denmark show that the mould most frequently occurring on building partitions is *Penicillium chrysogenum*, and among *Aspergillus*, *Aspergillus versicolor* [17]. This fact is confirmed by tests conducted in this paper.

On the basis of the mycological tests performed, it is possible, based on literature on the subject, to determine the potential hazard for humans inside the infected rooms. In 1996, a list was drawn up under the auspices of the European Confederation of Medical Mycology, which specified the classification of biosafety levels (BSL) for the respective species of fungi (BSL 1/3) [15].

According to this list, it is possible to specify species of moulds occurring in residential housing, which are pathogenic for humans and animals and which were most frequently present on building partitions in the Lubuskie province (Table 6).

The isolated moulds, including *A. versicolor*, are classified into the BSL1 category. This means that infections which may be caused by these species are most frequently benign, superficial, and non-invasive. In the rooms subjected to the tests, the most threatening pathogens were not present on the walls (BSL 3).

Moulds are the cause of many allergic diseases [15,16,17,58,59]. Allergens of moulds congested in badly ventilated rooms constitute a real threat for people with atopy [60]. It is known that the spores of *Aspergillus, Cladosporium*, and *Penicillium* present in damp buildings may cause asthma and/or rhinitis among atopic residents. It has been proved that raw extracts including *A. versicolor* may cause the release of histamines without the mediation of IgE in human cells of the mucous membranes from lungs in vitro [32]. Allergic reactions may be caused both by the spores and the fragments of mycelium. They are caused mainly by proteins, components of walls of moulds (β-(1-3)-d-glucans), microbial volatile organic compounds (MVOC) and mycotoxins, as well as other organisms which occur together with the moulds, such as mites (*Acari*) [10,61,62]. The respiratory system allergies are caused mainly by *Aspergillus* and *Penicillium* [16,17].

*Aspergillus versicolor* may cause allergic diseases. Among 82 species of moulds occurring in residential housing in the Lubuskie province, the other allergenic fungi include: *Penicillium chrysogenum, Aspergillus niger*, *Cladosporium herbarum*, *Alternaria alternata,* and *Aspergillus flavus*. The moulds which may occur with lower frequency in buildings, but still may cause allergic diseases, include *Penicillium glabrum*, *Aspergillus fumigatus*, and *Aureobasidium pullulans* (present paper).

At present, the time of exposure and the concentration of mycotoxins which may have a negative impact on the human health of residents is not known [63]. However, the presence of the moulds on building partitions may not be tolerated. It is necessary to carry out defungusing work in such rooms after the prior performance of mycological tests and structural surveys.

## 5. Conclusions 

The results of the toxicological research demonstrated that in order to assess the mycotoxic risk, it is possible to apply biotests, using the planarian *Dugesia tigrina* Girard instead of the costly physicochemical analysis by means of HPLC, which is additionally not friendly to the natural environment. This was confirmed by the results of the analyses carried out by means of HPLC. Strains of *A. versicolor*, which have the biological abilities to synthesise sterigmatocystin, occur on building partitions. However the number of highly mycotoxigenic strains in laboratory conditions is small. All of the research results presented in the paper are innovative as, owing to consequent complementation (the number of statistically significant tests), they led to valuable conclusions. The paper includes quotations from our own 17 research publications which were continued until our own two tables presented in this paper were drawn up (Table 3 and Table 4).

The mycological tests demonstrated the frequent occurrence of *Aspergillus versicolor* on inner building partitions (frequency 4). The conducted toxicological tests proved that *Aspergillus versicolor* is the most dangerous species for humans and animals in infected rooms owing to the synthesis of toxic and carcinogenic sterigmatocystin ST (category 2 B according to International Agency for Research of Cancer) in various quantities. All of the samples of air-dry mould biomass with the laboratory medium contained sterigmatocystin. *A. versicolor* is an allergenic species which may cause benign fungal infections. Biotoxicological tests of extracts from the biomasses of *A. versicolor* are necessary for the evaluation of the mycotoxic risk in buildings.

## Figures and Tables

**Table 1:** Toxicity classes of poisonous substances [54].

Quantity of Poison—Result of Toxicity Test (mg·L^−3^)	Toxicity Classes	Classes
<1	highly toxic	I
1–10	potently toxic	II
10–100	medium toxic	III
100–1000	weakly toxic	IV
>1000	barely toxic	V

**Table 2:** Toxicity classes of mould biomasses [13,33].

Quantity of Important Mycotoxins—Result of Chromatographic Analysis (mg·kg^−1^)	Toxicity Classes
≤1 mg·kg^−1^	non toxic
>1–10 mg·kg^−1^	weakly toxic
>10–100 mg·kg^−1^	potently toxic
>100 mg·kg^−1^	highly toxic

**Table 3:** Species of moulds isolated from building partitions in the area of the Lubuskie Province.

Types and Species of Moulds		Frequency in Flats *
*Absidia*	*corymbifera* Sacc. et Trotter	1
	*glauca* Hagem	1
*Acremonium*	*bacillisporum* W. Gams	1
	*charticola* W. Gams	1
	*murorum* W. Gams	1
	*strictum* W. Gams	3
*Alternaria*	*alternata* Keissler	2
	*tenuissima* Wiltshire	1
*Arthrinium*	*phaeospermum* M.B. Ellis	1
*Aspergillus*	*carbonarius* Thom	1
	*clavatus* Desmazieres	1
	*flavus* Link	3
	*fumigatus* Fressenius	1
	*niger* van Tieghem	3
	*terreus* Thom	1
	*ustus* Thom et Church	2
	*versicolor* Tiraboschi	4
*Aureobasidium*	*pullulans* Arnaud	1
*Beauveria*	*bassiana* Vuillemin	1
*Botrytis*	*cinerea* Persoon ex Fries	1
*Botryotrichum*	*piluliferum* Sacc. et Marchal	1
*Chaetomium*	*elongatum* Czerepanova	2
	*torulosum* Bainier	1
*Chromelosporium*	sp.	1
*Cladosporium*	*cladosporioides* de Vries	2
	*herbarum* Link ex Gray	5
	*macrocarpum* Preuss	3
	*sphaerospermum* Penzig	1
*Doratomyces*	*stemonitis* Pers.	1
*Epicoccum*	*nigrum* Link	1
*Fusarium*	*aquaeductum* Lagerheim	1
	*culmorum* Saccardo	1
	*equiseti* Saccardo	1
	*oxysporum* Schlechtendal ex Fries	1
	*sambucinum* Fuckel	1
	*solani* Saccardo	1
	*sporotrichioides* Sherbakoff	1
	*verticillioides* Nirenberg	1
*Geotrichum*	*candidum* Link	1
*Gilmaniella*	*humicola* Barron	1
*Humicola*	*brevis* Gilman et Abbott	1
	*fuscoatra* Traaen	1
*Monocillium*	*indicum* S. B. Saksena	1
*Moniliella*	*acetobutens* Stolk & Dakin	1
*Mucor*	*circinelloides* van Tieghem	1
	*globosus* Fischer	1
	*hiemalis* Wehmer	1
	*mucedo* Fresenius	1
	*piriformis* Fischer	1
	*plumbeus* Bonorden	1
	*racemosus* Fresenius	5
*Paecilomyces*	*farinosus* Brown et Smith	1
	*marquandii* Hughes	1
	*variotii* Bainier	1
*Penicillium*	*aurantiogriseum* Dierckx	1
	*brevicompactum* Dierckx	1
	*chrysogenum* Thom	5
	*expansum* Link ex Gray	1
	*funiculosum* Thom	1
	*glabrum* Westling	1
	*janthinellum* Biourge	1
	*lanosum* Westling	3
	*thomii* Maire	1
	*viridicatum* Westling	1
	*vulpinum* Seifert et Samson	1
	*waksmanii* Zaleski	1
*Phoma*	*glomerata* (Corda) Wollenw.	1
*Rhizomucor*	*pusillus* Schipper	1
*Rhizopus*	*stolonifer* Lind.	2
*Scopulariopsis*	*brevicaulis* Bainier	1
	*candida* Vuill.	1
*Stachybotrys*	*chartarum* Hughes	1
*Thamnidium*	*elegans* Link	2
*Trichoderma*	*koningii* Oudemans	1
	*viride* Persoon ex Gray	2
*Trichothecium*	*roseum* Link ex Gray	2
*Tritirachium*	*oryzae* (Vincens) de Hoog	1
*Ulocladium*	*botrytis* Preuss	2
	*chartarum* Simmons	4
	*consortiale* Simmons	1
*Verticillium*	*lecanii* Viégas	1
	*luteoalbum* Subramanian	2

* Frequency in flats: >0%–5% (1, sporadically), >5%–10% (2, individually), >10%–15% (3, fairly frequently), >15%–20% (4, frequently), >20% (5, very frequently) according to Piontek [22].

**Table 4:** Specification of the results of toxicological tests from biomasses of *A. versicolor*.

No.	Quantity of ST (mg·kg^−1^)	Classification According to Chełkowski [13,33]	LC 50 for *Dugesia tigrina* (mg·L^−3^)	Classification According to Liebmann [54]
1	<0.03	*non toxic*	n.t *	class IV *(weakly toxic)*
2	<0.03		n.t	
3	<0.03		n.t	
4	0.07		n.t	
5	1.28	*weakly toxic*	603	
6	1.30		399	
7	1.50		104.71	
8	2.69		150	
9	2.89		122	
10	6.48		132	
11	6.12		107	
12	7.20		105	
13	8.31		102	
14	13.6	*potently toxic*	70.9	class III *(medium toxic)*
15	15.1		25.1	
16	18.5		72.4	
17	28.2		26.8	
18	48.0		40.7	
19	54.2		20.7	
20	58.2		27.5	
21	63.2		67.6	
22	63.4		53.7	
23	63.5		51.3	
24	63.8		50.1	
25	63.9		10.9	
26	65.0		23.4	
27	78.3		18.0	
28	68.7		36.3	
29	84.0		29.0	
30	84.5		22.9	
31	85.7		20.8	
32	86.8		19.9	
33	88.9		31.0	
34	90.2		12.9	
35	94.1		10.7	
36	99.7		39.9	
37	107	*highly toxic*	28.0	
38	112		19.2	
39	120		19.7	
40	132		9.6	class II *(potently toxic)*
41	137		9.9	
42	148		9.1	
43	149		9.0	
44	273		9.1	
45	534		8.2	

* n.t—non toxic.

**Table 5:** Mycotoxins synthesised by selected moulds isolated from building partitions of analysed residential housing.

Species	Produced Mycotoxins [10,14,34] *
*Aspergillus versicolor*	**cyclopiazonic acid**, **sterigmatocystin**, **5–methoxysterigmatocystin**, dihydroxy–sterigmatocystin, nidulotoxin, averufin, versiconol, versicolorin A, B, C
*Penicillium chrysogenum*	**penicillic acid**, **rokefortin C**, **meleagrin**, fusigen, xantocilin, 6–amino–penicillic acid, negapilin, penicillin G, sideramin, chryzogin

* The important toxic metabolites are specified **in bold.**

**Table 6:** Classification of biosafety levels of selected moulds potentially pathogenic for humans and animals [15].

Species of Mould	BSL *
*Aspergillus versicolor*	1
*Cladosporium herbarum*	1
*Mucor racemosus*	1
*Penicillium chrysogenum*	1
*Ulocladium chartarum*	1

* BSL1—infections are superficial, non-invasive or benign. * BSL2—in patients with severe immunological disorders, moulds can cause deep opportunistic infections. * BSL3—pathogens potentially capable of inducing severe deep fungal infections in apparently healthy people [15].

## References

[1] Piontek M., Jasiewicz M., Bednar K. (2010). Mould biodeterioration caused by technological defects in dwelling buildings. Ochrona Przed Korozją.

[2] Piontek M., Jasiewicz M., Łuszczyńska K., Dudzińska M.R. (2011). Thermal Modernization and Biodeterioration of Prefabricated Elements of Buildings—A Case Study—W: Management of Indoor Air Quality.

[3] Piontek M., Bednar K. (2006). The increase in the number of moldy buildings by the presence of mold in a new building with modern technologies. Ochrona Przed Korozją.

[4] Gutarowska B., Piotrowska B. (2007). Methods of mycological analysis in buildings. Build. Environ..

[5] Flannigan B., Samson R.A., Miller J.D. (2011). Microorganisms in Home and Indoor Work Environments: Diversity, Health Impacts, Investigation and Control.

[6] Gaylarde C., Otlewska A., Cellikol-Aydi S., Skóra J., Sulyok M., Pielech-Przybylska K., Gillatt J., Beech I., Gutarowska B. (2015). Interactions between fungi of standard paint test method BS3900. Int. Biodeterior. Biodegrad..

[7] Dales R.E., Zwanenburg H., Burnett R., Franklin C.A. (1991). Respiratory health effects of home dampness and molds among Canadian children. Am. J. Epidemiol..

[8] Hendry K.M., Cole E.C. (1993). A review of mycotoxins in indoor air. J. Toxicol. Environ. Health.

[9] Johanning E., Biagini R., Hull D., Morey P., Jarvis B., Landsbergis P. (1996). Health and immunology study following exposure to toxigenic fungi (*Stachybotrys chartarum*) in water-damaged office environment. Int. Arch. Occup. Environ. Health.

[10] Piontek M. (2004). Moulds and Estimation of Mycotoxic Threat in Dwelling Buildings.

[11] Bloom E., Bal K., Nyman E., Must A., Larsson L. (2007). Mass spectrometry-based strategy for direct detection and quantification of some mycotoxins produced by Stachybotrys and *Aspergillus* spp. in indoor environments. Appl. Environ. Microbiol..

[12] Domsch K.H., Gams W., Anderson T.-H. (1980). Compendium of Soil Fungi. I, II.

[13] Chełkowski J. (1985). Mycotoxins, Mycotoxin-Producing Fungi and Mycotoxicosis.

[14] Samson R.A., Hoekstra E.S., Frisvad J.C. (2004). Introduction to Food and Airborne Fungi.

[15] De Hoog G.S., Guarro J. (2000). Atlas of Clinical Fungi.

[16] Flannigan B. (2001). Deteriogenic micro-organism in houses as a hazard to respiratory health. Int. Biodetrior. Biodegrad..

[17] Flannigan B. Microbial aerosols in buildings: Origin, health implications and controls. Proceedings of the II Conference on Microbial Biodegradation and Biodeterioration of Technical Materials.

[18] Grant C., Hunter C.A., Flannigan B., Bravery A.F. (1989). Water activity requirements of moulds isolated from domestic dwellings. Int. Biodeterior..

[19] Samson R.A., Houbraken J., Thrane U., Frisvad J.C., Andersen B. (2010). Food and Indoor Fungi.

[20] Gravesen S., Frisvad J.C., Samson R.A. (1994). Microfungi.

[21] Tuomi T., Reijula K., Johnsson T., Hemminki K., Hintikka E.L., Lindroos O., Kalso S., Koukila-Kähkölä P., Mussalo–Rauhamaa H., Haahtela T. (2000). Mycotoxins in crude building materials from water-damaged buildings. Appl. Environ. Microbiol..

[22] Piontek M. Moulds occurring in buildings of the Lubuskie province, Poland. Proceedings of the II Conference on Microbial Biodegradation and Biodeterioration of Technical Materiale.

[23] Engelhart S., Lock A., Skutlarek D., Sagunski H., Lommel A., Färber H., Exner M. (2002). Occurrence of toxigenic Aspergillus versicolor isolates and sterigmatocystin in carpet dust from damp indoor environments. Appl. Environ. Microbiol..

[24] Toepfer I., Mentlein R., Petersen K., Portner C., Butte W. (2008). Detection of Sterigmatocystin in House Dust.

[25] Jurjević Z., Peterson S.W., Solfrizzo M., Peraica M. (2013). Sterigmatocystin production by nine newly described *Aspergillus* species in section Versicolores grown on two different media. Mycotoxin Res..

[26] Piontek M. (1999). Moulds Atlas.

[27] Nielsen K.F. (2002). Mould Growth on Building Materials. Secondary Metabolites, Mycotoxins and Biomarkers. Ph.D. Thesis.

[28] Veršilovskis A., Bartkevičs V., Miķelsone V. (2008). Sterigmatocystin presence in typical Latvian grains. Food Chem..

[29] Cabaret O., Puel O., Botterel F., Pean M., Bretagne S., Delaforge M. (2011). Contribution of uniformly 13C-enriched sterigmatocystin to the study of its pulmonary metabolism. Rapid Commun. Mass Spectrom..

[30] Rank C., Nielsen K.F., Larsen T.O., Varga J., Samson R.A., Frisvad J.C. (2011). Distribution of sterigmatocystin in filamentous fungi. Fungal Boil..

[31] Nielsen K.F., Thrane U., Larsen T.O., Nielsen P.A., Gravesen S. (1998). Production of mycotoxins on artificially inoculated building materials. Int. Biodeterior. Biodegrad..

[32] Nielsen K.F., Gravesen S., Nielsen P.A., Andersen B., Thrane U., Frisvad J.C. (1999). Production of mycotoxins on artificially and naturally infested building materials. Mycopathologia.

[33] Chełkowski J., Wiewiórska M., Pietrzak J. (1984). Toxicity estimation of fungal biomasses with use *Artemia salina* shrimp larvae in biological test. Ferment. Fruit Veg. Process. Ind..

[34] Cole R.J., Cox R.H. (1981). Handbook of Toxic Fungal Metabolites.

[35] Pasanen P., Korpi A., Kalliokoski P., Pasanen A.-L. (1997). Growth and volatile metabolite production of *A. versicolor* in house dust. Environ. Int..

[36] International Assisted Reproduction Center (IARC) (1993). Some naturally occurring substances: Food items and constituents, heterocyclic aromatic amines and mycotoxins. International Agency for Research of Cancer. Monographs on the Evaluation of the Carcinogenic Risk of Chemicals to Humans.

[37] Hoekstra E.S., Samson R.A., Summerbell R.C., Samson R.A., Hoekstra E.S., Frisvad J.C. (2004). Methods for the detection and isolation of fungi in the indoor environments. Introduction to Food and Airborne Fungi.

[38] Piontek M. (2007). Strains of *Aspergillus versicolor* Tiraboschi synthesizing sterigmatocistin and the differentiation of mycotoxic risk dependent on their productivity in housing buildings. Mycotoxin Res..

[39] Piontek M. (2010). Use of planarian *Dugesia tigrina* Girard bioassay for assessing the toxicity of sterigmatocistin produced by *Aspergillus versicolor* Tiraboschi. Environ. Prot. Eng..

[40] Cooke W.B. (1963). A laboratory Guide to Fungi in Polluted Waters, Sewage and Sewage Treatment Systems.

[41] Barnett H.L. (1965). Illustrated Genera of Imperfect Fungi.

[42] Raper K.B., Fennel D.I. (1965). The Genus Aspergillus.

[43] Litvinov M.A. (1967). Opredelitel’ Mikroskopicheskikh Pochvennykh Gribov.

[44] Fassatiova O. (1983). Moulds in Technical Microbiology.

[45] Kwaśna H., Chełkowski J., Zajkowski P. (1991). Fungi (Mycota), Tom XXII; Fusarium.

[46] Pitt J.I. (2000). A Laboratory Guide to Common Penicillium Species.

[47] Piontek M., Łuszczyńska K., Solarz K. (2011). The occurence of mites (Acari) on walls infested with moulds in dwelling houses. Civil Environ. Eng. Rep..

[48] Piontek M. (1983/1984). The regenerative ability of the planarian *Dugesia tigrina* (Girard) and the possibility of its use in reproduction of this species. Acta Hydrobiol..

[49] Piontek M. (1998). Application of *Dugesia tigrina* Girard in toxicological studies of aquatic environments. Polskie Arch. Hydrobiol..

[50] Piontek M. (1999). Use of the planarian *Dugesia tigrina* Girard in studies of acute intoxication. Polskie Arch. Hydrobiol..

[51] Piontek M. (1999). Use of a planarian *Dugesia tigrina* Girard in the studies of acute toxicity of organic substances. Polskie Arch. Hydrobiol..

[52] Piontek M. Application of the *Dugesia tigrina* Girard bioassay in mycotoxicological investigations. Part I. Acute toxicity. Proceedings of the VII International Scientific Conference.

[53] Weber E., Fischer J.G. (1972). Grundriss der Biologischen Statistik für Naturwissenschaftler, Landwirte und Mediziner.

[54] Liebmann H., Fischer J.G. (1962). Handbuch der Frischwasser und Abwasserbiologie.

[55] ZFT/PB/06–12/Edition 1, Date of Issue 2011.04.28. The Determination of the Content of Sterigmatocistin in Fodder and Mycelium by Means of High-Performance Liquid Chromatography. http://www.piwet.pulawy.pl/piwet7/index_b.php?strona=zawar_9e.

[56] Piontek M. (2004). Moulds occurring in buildings of the Lubuskie province, Poland. Int. Biodeterior. Biodegrad..

[57] Buchmiet E., Żakowska Z. Toxicity of moulds on building materials. Proceedings of the V Conference on Microbial Biodegradation and Biodeterioration of Technical Materials.

[58] Baran E. (1998). Outline of Medical Mycology.

[59] Żukiewicz-Sobczak W., Sobczak P., Krasowska E., Zwoliński J., Chmielewska-Badora J., Galińska E.M. (2013). Allergenic potential of moulds isolated from buildings. Ann. Agric. Environ. Med..

[60] Bogacka E. (1997). Sick buildings. Mikol. Lek..

[61] Piontek M., Piontek R., Bednar K. (2004). Alergenic moulds in dwelling buildings. Mycotoxins and Pathogenic Moulds in the Environment: VII International Scientific Conference.

[62] Piontek M., Bednar K. (2006). Micotoxicity of Allergenic Moulds in Dwelling Buildings.

[63] Grajewski J., Twarużek M. (2004). The healthy aspects of the influence of moulds and mycotoxins. Alergia.

